# Knee Laxities Changes with Sex-steroids throughout the Menstrual Cycle Phases in Athlete and Non-athlete Females

**DOI:** 10.1055/s-0043-1771007

**Published:** 2024-03-21

**Authors:** Firouzeh Dehghan, Rahman Soori, Ashril Yusof

**Affiliations:** 1 Departamento de Ciências do Esporte, Campus Internacional de Kish, Universidade de Teerã, Ilha de Kish, Irã; 2 Departamento de Fisiologia do Exercício, Faculdade de Ciências do Esporte e Saúde, Universidade de Teerã, Teerã, Irã; 3 Departamento de Ciência do Exercício, Centro Esportivo, Universidade da Malásia, Kuala Lumpur, Malásia

**Keywords:** knee joint, menstrual cycle, hormone, sex steroid, relaxin, athletes

## Abstract

**Objective:** Our study investigated changes of knee laxities in athletes and non-athletes females and relationship between knee laxity and sex-steroid at menstrual cycle phases.

**Methods:** Forty six healthy females, twenty four athletes and twenty two non-athletes not on hormone contraceptive pills, had no previous knee injuries and with regular menstrual cycles for 3 consecutive months, participated in the study. Medial and lateral knee laxities were determined by varus-valgus tests at follicular, ovulatory and luteal phases. Serum level of relaxin, estrogen, progesterone and testosterone were determined by ELISA and radioimmunoassay.

**Results:** Knee laxities in athletes and non-athletes at 0° and 20° flexion were the highest in luteal phase with non-athletes possess greater laxity than athletes. Positive correlation between progesterone and relaxin levels with knee laxities were observed. Meanwhile, the levels of both hormones were highest in the luteal phase.

**Conclusion:** Increased medial and lateral knee laxities in athletes and non-athletes associated with high serum progesterone and relaxin levels in luteal phase may contribute toward increased risk of non-contact knee injury. However, lower knee laxity in athletes than non-athletes suggest that exercise could be a protective factor.

## Introduction

The most common injuries related to joints have been reported to involve the ankles and the knees. 1 Knee injuries which occur during sports is mostly associated to sub-luxation or dislocation. 2 A clear understanding on the injury pattern, the mechanisms underlying this injury and the risk factors is crucial in exercise physiology and sports medicine which could be related to gender, genetic and whole-body aerobic capacity. 3 Knee injuries undoubtedly affect the athlete performances that can prevent by increasing the strength of the quadriceps muscle during isometric, eccentric contractions, and concentric. 4 A remarkable number of non-traumatic injuries among women during sports over the years has led to multiple studies being performed to better understand the underlying mechanism involved. 5 6 Females are known to be more vulnerable than males toward knee injury. 7 In female, the occurrence was related to different phases of the menstrual cycle. 8 Several reports indicated that high incidence of non-contact knee injury happened during the follicular phase of the cycle, while others reported that the incidence is the highest at ovulation and in the luteal phase of the cycle. 9 10 These raised the possibility that female sex hormones could be involved in this injury. The role of sex steroids on knee injury remains poorly understood and represent an area for investigation.

Female were found has two to ten times greater risk of non-traumatic knee injury than male. 11 The reason for this occurrence is unclear. Evidences have suggested that female sex hormones may be involved as they could affect knee laxity and knee laxity increase during the menstrual cycle. 12 Up-to-date, the reports with regards to sex steroids effect on knee laxity were conflicting. Several studies indicated that female anterior knee laxity was highest in follicular (pre-ovulatory) phase, 13 while others reported that laxity was the greatest in ovulatory, 12 and luteal phases. 14 15 There were studies which showed that no differences in knee laxity was observed between phases of the menstrual cycle. 16 In addition to sex steroids, relaxin was also found to influence knee laxity where high relaxin levels could contribute to increased knee laxity. 15

While previous studies focused mainly on sex-steroid effect on ACL laxity, 6 17 changes in laxity of LCL and MCL under sex-steroid influence remain unknown. Furthermore, the relationship between serum sex-steroids and relaxin levels with medial and lateral knee laxities has never been identified. In view of this, our study aimed at investigating changes in medial and lateral knee laxities in females particularly in athletes at different phases of menstrual cycle. The relationship between laxities and serum sex-steroid and relaxin levels will also be determined. We hypothesized that medial and lateral laxities of the knee changes with phases of the menstrual cycle and increasing in luteal phase, therefore contributed toward differences in the incidence of non-contact knee injuries between menstrual phases. Meanwhile changes in non-female athletes will also be investigated.

## Methods

### Subject's Recruitment

Forty six healthy females, 24 professional athletes (20.3 ± 1.28 years, 21.9 ± 2.6 BMI) with over 6–7 years experience of basketball, netball, footsul, swimming, and handball national team members, and 22 non-athletes (21.7 ± 2.27 years, 22.2 ± 3.42 BMI) who were not on hormonal contraceptives and regular cycles for 3 consecutive months volunteered to participate in this study. The inclusion criteria include no history of knee surgery or history of injury or chronic pain in both lower extremities for the past 1 year. Subjects were not on any specific medications. Amenorrheic, oligomenorrhoeic and polymenorrhoeic subjects were excluded. We also excluded subjects who had undergone surgery, leg trauma, as well as those who had no regular menstrual cycle. The participants were informed detail of the study from the information sheet provided, and written informed consent was also obtained. This study was registered (Medical Ethics Number 1010.90) and approved by the ethical review of the Medical Centre Board, University Malaya. The methods were performed in accordance with the approved guidelines of institutional Medical Centre Board ethics committee.

### Determination of Basal Body Temperature (BBT) and Phases Determination

**Fig. 1:**
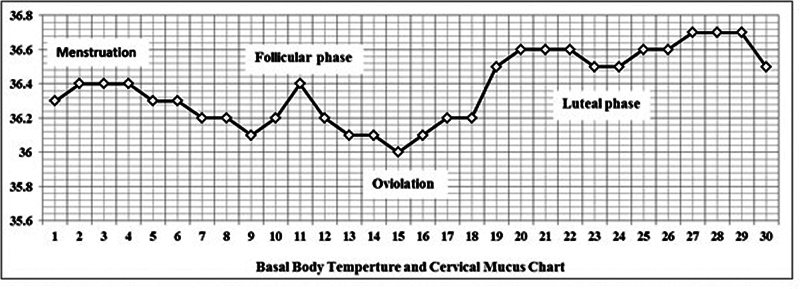
Basal body temperatures chart.

The basal body temperature (BBT) was used to identify the ovulatory cycle which was featured by a slight increase in core body temperature (∼0.5oC) during mid-cycle. 18 BBT was measured when the body was at rest. Based on BBT test, subject's individual phases were determined. In determining BBT, rectal temperature (ADC ADTemp V Fast Read Pen Type Digital thermometer, American Diagnostic, USA) was obtained. The temperature was taken at 7 o'clock in the morning for 1 month duration. All subjects participated in this study were found to have ovulatory cycle. The sample of BBT recorded is shown in Fig. 1 .

### Body Composition Measurement by Bioelectrical Impedance Analysis (BIA)

BIA is a reliable and accessible method for screening body fat. The standing BIA device (BC-418, Tanita Corp., Tokyo, Japan), with stainless-steel plates is used to measure whole body and segmental impedance. Subjects stand without shoes on the base and hold the handgrips with embedded electrodes; soles of both feet are in contact with the electrode plates. They were weighed, and impedance was measured their total body water, total protein, total mineral, skeletal muscle mass, body fat mass, body mass index, waist hip ratio, fitness score (estimated fitness score based on total body condition) and basal metabolic rate in athletes and non-athletes (no clicking on the machin whether subject is an athlete or not). Measurements were performed in the morning before breakfast (7:30–8:30 am ) at the same day of sampling by the same examiner in all subjects. The participants were told not to eat or drink before the measuremen. In the 24 hours prior to the measurements they also did not consume any medications (including alcohol and caffeine) or perform high intensity physical activity that could effect the results.

### Blood Sample Collection and Serum Hormones Analyses

Blood samples were collected three times in a month: follicular phase, ovulatory phase, and mid-luteal phase. 19 The three phases were determined by using a fertility chart and at the days stated, blood samples were withdrawn via venepuncture. The blood was collected in a separator tube (SST), allowed to clot at room temperature for 30 minutes. The clotted blood was then centrifuge at 3000 g, 15 minutes. Serum samples were then aliquot and stored at –20°C. Serum samples were analyzed in duplicate for relaxin concentration by using specific human relaxin peptide enzyme-linked immunosorbent assay (ELISA) kit (CUSABIO – USA, with detect range of 6.25 pg/ml-400 pg/ml. ELISA was performed according to the manufacturer's guidelines. The absorbance for relaxin was determined by using a microplate reader (iMark; Bio - Rad, Hercules, CA, USA) at a wavelength of 450 nm. A set of standard serial dilutions of known concentrations of relxin were provided by the manufacturer and were used to construct a standard curve to determine the hormone levels (400, 200, 100, 50, 25, 12.5, 6.25, 0 pg/ml). Meanwhile, radioimmunoassay (RIA) was used to determine serum levels of estrogen in pg/ml, progesterone in ng/ml and testosterone Radioimmunoassay (RIA) was used to determine serum levels of estrogen in pg/ml, and progesterone in ng/ml.

### Knee Rotational Movement

Varus and valgus stress tests were applied to the knee to determine medial and lateral laxities. Measurements were performed manually by using a orthopaedic goniometer (model: MR0104 PVC material 180°), and repeated to confirm by using an custom-made electronic chair which was designed based on described previously. 20 To establish reliability, examination was performed by two raters (a physical therapist and a human movement scientist), both trained by a clinician, independently performed measurements. The examiners were blinded to phase of subjects cycle. Subjects' both knees were assessed for changes in varus/valgus angles. The test was accomplished by fixing the knee at 0° and 20° of flexion. Ligament injuries have not observed the presence of varus/valgus or axial load up to 20°. 21 In view of that the angle of 0° and 20° are chosen for this study. All procedures were identical to those described in the clinical examination textbook, 22 and van der Esch et al. 20

The varus/valgus measurements were conducted manually by using a orthopaedic goniometer. Varus test is a stress force applied from medial side by adducting the ankle. Testing the LCL was performed when the knee was flexed at 0° or 20° of flexion with the subjects lying flat. One hand of the examiner was placed over the lateral joint line while another hand held the lower leg firmly at the ankle. Meanwhile, valgus test is a stress force applied on the lateral side by abducting the ankle. Stretching of MCL was performed when the knee was at 0° or 20° of flexion. When performing this test, patient was lying flat with one examiner's hand placed over the medial joint line and another hand held the lower leg firmly. The diffrences of angle was recorded manually by using an orthopaedic goniometer.

The varus/valgus measurements were reconducted by using an custom-made electronic chair. Reliability ranging of intraclass correlation coefficient (ICC) test was performed to detect minimal differences in varus and valgus deviations or high precision measurement with high reproducibility. The reliability was conducted on ten subjects of athletes and non athletes, with reliability an scores ranging of 0.95 and 0.93 (ICC) respectively. The females participants were seated comfortably in the electronic chair with a back support. The knee joint was fixed in 0° or 20° of flexion throughout the measurement. The thigh, lower leg and ankle were fixed to the chair at 0° or 20° and medial, lateral, internal, external rotational movements in leg were not possible. The subject's foot and distal part were fastened using clamps at the ankle or distal part of the leg. The lower leg and upper thigh were fastened to the device using a Velcro bandage. A free moving arm was directly located under the tibiofemoral subject's joint as a axis of rotation (middle of the popliteal fossa). To supply a steady moment to the knee of 7.7 Nm, a deadweight was used which the weight was attached by a cord to the freemoving arm. The cord was fastened 74cm from the axis of rotation of the arm. The weight load was applied to both medially and laterally sides of the lower leg, resulted in the knee joint varus/valgus measure. Raters were seated behind the subjects and applied the load slowly to their lower leg by hand in a standardized manner. An attached digital measurement system was recorded the end point of the varus/valgus rotaitional movement during 5s. To avoid increasing of muscle tone resulting from pain during the measurement, subjects were trained to relax report the onset of pain. Laxity movement of the knee joint angle was calculated as the sum of the varus/valgus deviations in degree. 23 24 The result of manual laxity tests was almost the same as for electronic chair of the knee.

### Statistical Analysis

All data were presented as mean ± standard deviation. Hormone levels and rotational angles of each subject at different phases of the menstrual cycle were analyzed by descriptive statistics. Levene's equality of variable assumption was applied and the results revealed no significant differences among the observations. The means of these observations were computed and used for data analysis. Two-way analysis of variance (ANOVA) was used to compare between athletes and non-athletes groups. For variables with normal distribution, Pearson correlation coefficient and for non-normal distributed variable, Spearman correlation coefficient was applied. SPSS 18.0 were used in this study and *p* < 0.05 was considered as statistically significant.

## Results

### Body Composition Analyses

Differences between body composition in athletes and non-athletes at different phases of the menstrual cycle are shown in Table 1 . The average total body water, total protein, total mineral, skeletal muscle mass, body fat mass, body mass index, waist hip ratio, fitness score and basal metabolic rate in athletes and non-athletes were presented. Measurement was performed three times and average values were entered as a final measurement. Levene's equality of variable assumption stated that there were no significant difference between these variables. 25

**Table 1:** Characteristics and demographics of the subjects at different phases of the menstrual cycle

Variables	Athletes Mean ± SD	Non-Athletes Mean ± SD
**Age (years)**	20.3 ± 1.28	21.7 ± 2.27
**Height (cm)**	163 ± 2.75	158.5 ± 5.28
**Weight (kg)**		
- **Follicular phase**	58 ± 7.93	56.5 ± 9.96
- **Ovulatory phase**	57.5 ± 7.75	55.7 ± 9.99
- **Luteal phase**	58 ± 7.93	56.1 ± 10
**Total Body Water (kg)**		
- Follicular phase *	30.8 ± 2.42	27 ± 3.19
- Ovulatory phase *	30.4 ± 2.33	27 ± 3.1
- Luteal phase*	30.6 ± 2.45	27.1 ± 3.2
**Total Protein (kg)**		
- Follicular phase *	7.18 ± 0.75	8.24 ± 0.66
- Ovulatory phase *	7.21 ± 0.81	8.24 ± 0.66
- Luteal phase*	7.21 ± 0.85	8.22 ± 0.65
**Total Mineral (kg)**		
- Follicular phase *	3.08 ± 0.25	2.73 ± 0.32
- Ovulatory phase *	3.03 ± 0.26	2.69 ± 0.3
- Luteal phase*	3.05 ± 0.27	2.71 ± 0.33
**Skeletal Muscle Mass (kg)**		
- Follicular phase *	23 ± 2.05	19.7 ± 2.55
- Ovulatory phase *	22.7 ± 2.02	19.7 ± 2.47
- Luteal phase*	22.8 ± 1.91	19.8 ± 2.58
**Body Fat Mass (kg)**		
- Follicular phase *	15.7 ± 5.34 (26.7%)	19.1 ± 7.02(33.4%)
- Ovulatory phase *	15.9 ± 5.44 (26.7%)	19 ± 7.34 (33%)
- Luteal phase*	16.1 ± 5.5 (27.3%)	19 ± 7.06 (33%)
** Body Mass Index (kg/m ^2^ ) **		
- **Follicular phase**	21.9 ± 2.55	22.2 ± 3.37
- **Ovulatory phase**	21.8 ± 2.59	22.2 ± 3.51
- **Luteal phase**	22 ± 2.67	22.2 ± 3.39
Waist Hip Ratio*	0.82 ± 0.04	0.84 ± 0.06
Fitness Score (points)*	75.1 ± 2.93	69.3 ± 5.51
**Basal Metabolic Rate (kcal)**		
- Follicular phase *	1284 ± 75.8	1160 ± 96.6
- Ovulatory phase *	1272 ± 75	1167 ± 90.9
- Luteal phase*	1275 ± 77.2	1170 ± 94.7

Data showed the mean ± standard deviation for the impedance measurements. There are statistically significant differences in most of variables between athletes and non-athletes group as compared (*p < 0.05).

*p < 0.05 comparison between athlete/non-athlete groups.

Our findings indicated that mean weight of athletes were higher than non-athletes however the differences were not statistically significant. The weight was higher in luteal and menstruation phases as compared with follicular phase. Athletes in general had higher total body water as compared with nonathletes. However, there were no significant differences in total body water between menstrual cycle phases. Meanwhile, total protein estimate was significantly higher in non-athletes as compared with athletes ( *p* < 0.05) and no significant difference was noted between phases of the menstrual cycle. Total mineral content in athletes was significantly higher than non-athletes and in both groups, total mineral content was highest in menstruation phase of the cycle.

Skeletal muscle mass was higher in athletes as compared with non-athletes ( *p* < 0.05) and no significant difference was noted between phases of the menstrual cycle. Body fat mass was higher in nonathletes than athletes ( *p* < 0.05) and no significant difference was noted between phases of the menstrual cycle. Body mass index was slightly lower in athletes than non-athletes with no significant difference was noted between the two groups. Waist hip ratio was significantly lower in athletes than non-athletes, while fitness score index was significantly higher in athletes than non-athletes ( *p* < 0.05). Finally, athletes have higher basal metabolic rate (BMR) than non-athletes, however no significant difference in BMR was noted between phases of the menstrual cycle.

### Knee Joint Angles at Different Phases of the Menstrual Cycle

Table 2 and 3 show the degree of knee angles at 0° and 20° flexion in varus and valgus stress tests in athletes and non-athletes. In varus 0° and 20° of flexion tests, non-athletes have higher knee angle as compared with athletes at all phases of the cycle. In both groups, knee angle was the highest in the luteal phase, followed by follicular phase and the lowest in the ovulatory phase. In valgus 0° and 20° of flexion tests, non-athletes appear to have greater knee angle in all phases of the cycle as compared with athletes ( *p* < 0.05). The highest angle was noted in the luteal phase, followed by follicular phase and ovulatory phase.

**Table 2:** Knee joint angles of athletes and non-athletes (manually using an orthopaedic goniometer)

	Athletes Mean(SD)	Non-Athletes Mean (SD)	t value	P value	Effect size
**Varus 0°**					
- **Follicular phase**	3.75 (1.13)	5.33(1.02)	4.63	<0.05	1.47
- **Ovulatory phase**	1.80 (0.43)	3.44(0.95)	7.00	<0.05	2.38
- **Luteal phase**	5.45 (1.01)	7.19(1.01)	5.46	<0.05	1.72
**Valgus 0°**					
- **Follicular phase**	3.09 (1.21)	4.76(0.89)	4.99	<0.05	1.60
- **Ovulatory phase**	1.39 (0.51)	3.44(0.56)	12.09	<0.05	3.83
- **Luteal phase**	4.76 (1.32)	5.56(0.72)	2.37	<0.05	0.78
**Varus 20°**					
- **Follicular phase**	11.48(1.21)	12.80(1.03)	3.74	<0.05	1.18
- **Ovulatory phase**	9.35(0.75)	10.95(0.72)	6.89	<0.05	2.18
- **Luteal phase**	14.25(1.18)	15.11(1.05)	2.44	<0.05	0.77
**Valgus 20°**					
- **Follicular phase**	9.58(1.56)	11.71(1.51)	4.40	<0.05	1.39
- **Ovulatory phase**	7.63(1.00)	10.11(0.88)	8.36	<0.05	2.64
- **Luteal phase**	11.64(1.29)	12.98(1.79)	2.73	<0.05	0.87

Knee joint varus/valgus angles on 0 and 20 **°** of athletes and non-athletes are presented as mean ± standard deviation. A Statistically significant differences were found between athletes and non-athletes ( *P* < 0.05). Results indicating non-athletes had higher knee angle as compared with athletes at three different phases of the menstrual cycle; menstruation, follicular and luteal phases. Effect size is > 0.8.

**Table 3:** Knee joint angles of athletes and non-athletes (using an custom-made electronic chair to confirm)

	Athletes Mean(SD)	Non-Athletes Mean (SD)
**Varus 0°**		
- **Follicular phase**	3.88 (1.57)	5.52(1.77)
- **Ovulatory phase**	1.49 (0.71)	4.01(1.53)
- **Luteal phase**	4.95 (1.32)	6.97(1.68)
**Valgus 0°**		
- **Follicular phase**	3.22 (1.18)	4.64(0.52)
- **Ovulatory phase**	1.49 (1.32)	3.81(1.62)
- **Luteal phase**	4.69 (1.76)	6.03(1.25)
**Varus 20°**		
- **Follicular phase**	10.81(3.02)	11.98(2.11)
- **Ovulatory phase**	9.73(2.41)	10.83(1.56)
- **Luteal phase**	13.55(3.12)	14.87(1.55)
**Valgus 20°**		
- **Follicular phase**	9.85(1.56)	11.59(2.31)
- **Ovulatory phase**	7.71(2.07)	10.67(1.05)
- **Luteal phase**	12.02(2.14)	12.69(2.13)

### Changes in Serum Sex-Steroids and Relaxin Levels at Different Phases of the Menstrual Cycle

Table 4 shows the values of serum sex-steroids and relaxin levels in athletes and non-athletes. Our findings indicate that the highest estrogen level was observed in the ovulatory phase in both groups whereby there were no significant differences between athletes and non-athletes. Meanwhile, progesterone levels were the highest in luteal phase with athletes having a significantly lower level than non-athletes ( *p* < 0.05). Similarly, progesterone level was also higher in non-athletes as compared with athletes at follicular phase, although this level was ∼12 times lower than in the luteal phase of the cycle. No significant difference in progesterone level was observed in the ovulatory phase between the two groups.

**Table 4:** Sex-steroids and relaxin levels at different phases of the menstrual cycle

	Athletes Mean(SD)	Non-Athletes Mean(SD)	t value	P value	Effect size
**Estrogen (pg/ml)**					
- **Follicular phase**	170.20(39.86)	197.65(71.97)	1.49	>0.05	0.50
- **Ovulatory phase**	507.35(161.52)	492.30(276.96)	0.21	>0.05	0.07
- **Luteal phase**	439.50(150.54)	409.45(163.89)	0.60	>0.05	0.19
**Progesterone (ng/ml)**					
- **Follicular phase**	2.28(1.00)	4.19(2.53)	3.14	<0.05 **	1.08
- **Ovulatory phase**	1.80(1.24)	1.74(0.60)	0.20	>0.05	0.07
- **Luteal phase**	24.74(11.34)	31.33(8.09)	2.12	<0.05 **	0.68
**Testosterone (ng/ml)**					
- **Follicular phase**	0.82(0.38)	1.24(0.47)	3.08	<0.05 **	0.99
- **Ovulatory phase**	1.21(0.54)	1.39(0.48)	1.12	>0.05	0.36
- **Luteal phase**	1.22(0.61)	1.37(0.63)	0.76	>0.05	0.24
**Relaxin (pg/ml)**					
- **Follicular phase**	2.10(0.56)	1.69(1.27)	1.33	>0.05	0.45
- **Ovulatory phase**	1.38(0.87)	0.34(0.45)	4.73	<0.05 **	1.58
- **Luteal phase**	15.58(5.36)	10.35(2.96)	3.82	<0.05 **	1.26

Sex-steroids and relaxin levels are presented at different phases of the menstrual cycle as mean /standard deviation.** *p* < 0.05, (comparison between athletes and non-athletes). Effect size is > 0.8.

Meanwhile, the levels of testosterone, which were lower than estrogen and progesterone, were found to be higher in non-athletes than athletes. In non-athletes, testosterone levels were the highest in ovulatory phase followed by luteal phase. The lowest testosterone levels were noted in athletes at follicular phase. Serum levels of relaxin were highest in athletes as compared with non-athletes, in particular during the ovulatory and luteal phases of the cycle. No significant differences in relaxin levels were noted during the follicular phase between the two groups.

### Correlations between Sex-Steroids and Relaxin Levels with Knee Joint Angles

Table 5 , 6 and 7 show correlation between sex-steroid levels and knee joint angles in athlete and nonathlete females. In general, strong correlations were observed between progesterone and relaxin levels with knee joint angles in both varus and valgus tests (0° and 20° of flexion), in athletes and non-athletes. The highest correlations between serum levels of progesterone and relaxin with knee angles were observed in varus test at 20° of flexion in both athletes and non-athletes.

**Table 5:** Correlation coefficient between sex steroid and knee angles

	0 ^o^		20 ^o^	
	varus	valgus	varus	valgus
**Estrogen** - **Pearson**	-0.15	-0.17	-0.09	-0.14
**Progesterone** - **Spearman**	0.70**	0.62**	0.76**	0.62**
**Testosterone** - **Pearson**	0.07	0.12	0.12	0.07
**Relaxin** - **Spearman**	0.58**	0.50**	0.65**	0.47**

**Table 6:** Correlation coefficient of sex steroid and knee angles in athletes

	0 ^o^		20 ^o^	
	varus	valgus	varus	valgus
**Estrogen** - **Pearson**	-0.13	-0.15	-0.04	-0.14
**Progesterone** - **Spearman**	0.66**	0.58**	0.70**	0.59**
**Testosterone** - **Pearson**	0.05	0.03	0.06	-0.06
**Relaxin** - **Spearman**	0.69**	0.65**	0.74**	0.64**

**Table 7:** Correlation coefficient of sex steroid and knee angle in non-athletes

	0 ^o^		20 ^o^	
	varus	valgus	varus	valgus
**Estrogen** - **Pearson**	-0.17	-0.26*	-0.13	-0.17
**Progesterone** - **Spearman**	0.72**	0.65**	0.80**	0.60**
**Testosterone** - **Pearson**	-0.10	0.01	0.01	0.00
**Relaxin** - **Spearman**	0.81**	0.73**	0.80**	0.62**

* Correlation is significant at the 0.05 level.

** Correlation is significant at the 0.01 level.

## Discussion

The major findings from this study are (i) athletes have lesser degree of medial and lateral knee laxities as compared with non-athletes, (ii) both athletes and non-athletes have greatest medial and lateral knee laxities in luteal phase of the cycle (iii) progesterone and relaxin levels in both athletes and non-athletes were highest in luteal phase whereas estrogen levels were highest in ovulatory phase (iv) progesterone levels in luteal and follicular phases were lower in athletes as compared with nonathletes and (v) strong correlation was observed between serum progesterone and relaxin levels with medial and lateral knee laxities (both varus and valgus at 0 and 20°) in both athletes and non-athletes. Other findings are the athletes have higher total body water, total mineral content, skeletal muscle mass, fitness score and BMR as compared with non-athletes. The total protein content, body fat mass and waist to hip ratio in athletes were however lower than non-athletes.

We have found that medial and lateral knee laxities were highest in the luteal phase which correlate with highest serum progesterone and relaxin levels. The highest medial and lateral laxities could predispose to knee instabilities, therefore increasing the risk of knee dislocation and non-contact knee injuries for example ligament and menisceal tear. Previous reports have revealed that anterior laxity of the knee increases with increasing progesterone levels. 26 Higher incidences of ACL tear and non-contact knee injury were reported in the post-ovulatory or luteal phases where progesterone levels were high. 8

Recently, anterior laxity and valgus movement of the knee were reported to correlate with blood progesterone levels. 27 In animal for example rats, increased in collateral ligament laxities has been reported higher at diestrus and proestrus phases correlated with the high serum progesterone levels. 28 Evidence has suggested that progesterone influence on knee laxities could be due to increased collagen breakdown as the serum levels of biomarkers for collagen degradation were high in early luteal phase of menstrual cycle as reported in eumenorrhoeic women, 29 Our findings in athletes which indicate increased knee laxity in the luteal phase, that correlates with the high serum progesterone levels are in agreement with the finding by Heitz et al, 30 who reported that a significant increase in ACL laxity occur at peak progesterone levels in physically active women.

In this study, serum relaxin levels were found to correlate with knee laxity. The serum level of relaxin were highest in luteal phase parallel with the increased in serum progesterone levels. This was consistent with a report in female athletes where the level of serum relaxin positively correlates with serum level of progesterone. 31 In athletes, serum level of relaxin during ovulatory and luteal phase was ∼4.0 and 1.5 times higher respectively as compared with nonathletes. In athletes, serum relaxin levels were highest in the luteal phase (15.58 ± 5.36pg/ml) suggesting that relaxin might influence medial and lateral knee laxities. Therefore, increased in serum relaxin level could contribute to increased medial and lateral knee laxities as observed in athletes and non-athletes during the luteal phase of the cycle. Our findings were supported by a study by Dragoo et al., 32 (2011) who reported that serum relaxin levels strongly correlates with the incidence of ACL tear in elite collegiate female athletes. Meanwhile, in animal studies, relaxin administration to guinea pigs resulted in increased ACL laxity. 32 The increase in laxity could be due to several factors which include up-regulation of matrix metalloproteases (MMPs), 33 that cause increased collagen breakdown. 34 In addition, progesterone could also enhance relaxin action via up-regulating the expression of relaxin receptor isoforms RXFP1 and RXFP2 in knee collateral ligaments and patellar tendon of rats. 35 Relaxin receptor was also expressed in ACL of rodents, 36 and carpometacarpal joints of humans. 37

In this study, no significant difference was observed in estrogen and testosterone levels between athletes and non-athletes in both luteal and ovulatory phases of the menstrual cycle. In athletes, serum testosterone level was slightly lower in the follicular phase. Although serum estrogen levels were highest in ovulatory phase, laxity appeared to be the lowest. Therefore, we concluded that high estrogen level may not be responsible for the increase in medial and lateral knee laxities. Our findings were in contrast with several other reports which indicate that high serum estrogen levels were responsible for the increased in incidence of non-contact knee injury during ovulatory phase 12 38 39 The differences between our findings and others were unclear. Meanwhile, lower knee laxity in ovulatory phase suggested that high estrogen levels could decrease the risk of non-contact knee injury which was in contrast to high serum progesterone levels which caused the opposite effect. Our findings supported those of Wojtys et al, 8 who reported that significantly fewer non-traumatic knee injuries was observed in follicular phase as compared with post-ovulatory phase. Alternatively, lower risk of non-traumatic knee injury in the ovulatory phase may be due to lower serum progesterone and relaxin levels.

In this study, testosterone may not have influence on medial and lateral knee laxities as no positive correlation were observed between serum testosterone level and knee parameters. We have found that no significant difference in serum testosterone levels was noted between athlete and non-athlete in ovulatory and luteal phases of the menstrual cycle. A recent finding by O'Leary et al., 40 reported that prolonged aerobic exercise in women with normal menstrual cycle could induce a short-term elevation of plasma testosterone level. This effect however was not observed in our study, meanwhile reported that testosterone effect on knee laxity could be masked by the effect of estrogen and progesterone.

In conclusion, we have demonstrated that changes in medial and lateral knee laxities in athletes and non-athletes differ with menstrual cycle phases. We have shown that serum sex-hormones, in particular progesterone have strong positive correlation with medial and lateral knee laxities. Wiertsema et al, 41 reported that clinical test for example Lachman is reliable in determining the anterior–posterior laxity of ACL. Arthrometer could be used to determine anterior tibio-femoral movement however this device has limited application when measuring the medial and lateral knee laxities. The observed decreased in knee laxity in athletes could be due to greater muscular control as athletes have greater muscle mass, fitness level, and differences in the biomechanical properties in the ligament due to difference in loading. 42 The greater muscle mass would contribute toward greater tone which resists knee joint movement. The higher medial and lateral knee laxities in non-athletes especially in luteal phase suggest that this group are more susceptible toward non-traumatic injury involving the knee.

## Conclusion

The findings from this study is importance to the field of sports physiology and medicine as it could provide the basis underlying increased incidence of non-traumatic knee injury observed in females during the luteal phase of menstrual cycle. In view of this, precautions are needed to reduce the risk of non-traumatic knee injury in female athletes during this cycle phase.
