# Supplementary material for: Knee Laxities Changes with Sex-steroids throughout the Menstrual Cycle Phases in Athlete and Non-athlete Females
Source: Rev Bras Ortop (Sao Paulo). 2024 Mar 21;59(1):e29–37. doi: 10.1055/s-0043-1771007 (PMC10957278; doi:10.1055/s-0043-1771007)
Supplement: Related texts

# Research article: Mudanças na flacidez do joelho com esteroides sexuais durante as fases do ciclo menstrual em mulheres atletas e não atletas

**Keywords:** articulação do joelho, ciclo menstrual, hormônio esteroide sexual, relaxina, atletas

## Abstract

**Objetivo:** Nosso estudo investigou alterações na lassidão do joelho em atletas e não atletas do sexo feminino e a relação entre a lassidão do joelho e esteroides sexuais nas fases do ciclo menstrual.

**Métodos:** Quarenta e seis mulheres saudáveis, vinte e quatro atletas e vinte e duas não atletas, sem uso de pílulas anticoncepcionais hormonais, sem lesões anteriores no joelho e com ciclos menstruais regulares por 3 meses consecutivos, participaram do estudo. A lassidão medial e lateral do joelho foi determinada por testes de varo-valgo nas fases folicular, ovulatória e lútea. Os níveis séricos de relaxina, estrógeno, progesterona e testosterona foram determinados por ensaio imunoenzi mático (ELISA) e radioimunoensaio.

**Resultados:** A lassidão do joelho em atletas e não atletas em 0° e 20° de flexão foi maior na fase lútea; as não atletas apresentavam maior lassidão do que as atletas. Houve uma correlação positiva entre os níveis de progesterona e relaxina e a lassidão do joelho. Além disso, os níveis desses dois hormônios foram maiores na fase lútea.

**Conclusão:** O aumento da lassidão medial e lateral do joelho em atletas e não atletas, associado a altos níveis séricos de progesterona e relaxina na fase lútea, pode contribuir para o aumento do risco de lesão sem contato no joelho. No entanto, a menor lassidão do joelho em atletas do que em não atletas sugere que o exercício pode ser um fator protetor.

## Introdução

As lesões articulares são mais comuns nos tornozelos e joelhos. 1 As principais lesões de joelho associadas a práticas esportivas são as subluxações e luxações. 2 O entendimento claro sobre o padrão de lesão, os mecanismos subjacentes e os fatores de risco são cruciais na fisiologia do exercício e na medicina esportiva. Estes elementos podem estar relacionados ao sexo, à genética e à capacidade aeróbica de todo o corpo. 3 As lesões no joelho afetam o desempenho de atletas, que podem preveni-las aumentando a força do músculo quadríceps durante contrações isométricas, excêntricas e concêntricas. 4 Ao longo dos anos, o número notável de lesões não traumáticas entre mulheres que praticam esportes levou à realização de diversos estudos para compreender melhor o mecanismo subjacente. 5 6 As mulheres são sabidamente mais vulneráveis às lesões de joelho do que os homens. 7 Nas mulheres, essas lesões foram relacionadas às diferentes fases do ciclo menstrual. 8 Vários relatos indicaram que a alta incidência de lesões sem contato no joelho ocorreu durante a fase folicular do ciclo menstrual, enquanto outros indicaram uma maior incidência na ovulação e na fase lútea do ciclo. 9 10 Isso levantou a possibilidade de que os hormônios sexuais femininos pudessem estar envolvidos nessa lesão. O papel dos esteroides sexuais na lesão do joelho ainda é pouco entendido e representa uma área de investigação.

Nas mulheres, o risco de lesão não traumática do joelho é duas a dez vezes maior do que em homens. 11 O motivo disso não é claro. As evidências sugerem a possível participação de hormônios sexuais femininos, que podem influenciar a lassidão do joelho e aumenta-la durante o ciclo menstrual. 12 Até agora, os relatos sobre o efeito dos esteroides sexuais na lassidão do joelho foram conflitantes. Vários estudos indicaram que, em mulheres, a lassidão anterior do joelho era maior na fase folicular (pré-ovulatória), 13 enquanto outros relataram que a lassidão era maior na fase ovulatória 12 e na fase lútea. 14 15 Alguns estudos mostraram que não há diferenças na lassidão do joelho entre as fases do ciclo menstrual. 16 Além dos esteroides sexuais, descobriu-se também que a relaxina influencia a lassidão do joelho e que altos níveis desta molécula podem contribuir para o aumento da lassidão do joelho. 15

Embora estudos prévios enfocassem principalmente o efeito de esteroides sexuais na lassidão do ligamento cruzado anterior (LCA), 6 17 as alterações na lassidão do ligamento colateral lateral (LCL) e do ligamento colateral medial (LCM) sob influência de esteroides sexuais ainda são desconhecidas. Além disso, a relação entre os níveis séricos de esteroides sexuais e relaxina e a lassidão medial e lateral do joelho nunca foi identificada. Diante disso, nosso estudo teve como objetivo investigar alterações na lassidão medial e lateral do joelho no sexo feminino, principalmente em atletas em diferentes fases do ciclo menstrual. A relação entre a lassidão e os níveis séricos de esteroides sexuais e relaxina também será determinada. Nossa hipótese é que a lassidão medial e lateral do joelho muda conforme as fases do ciclo menstrual e aumenta na fase lútea, contribuindo para diferenças na incidência de lesões sem contato no joelho entre as fases menstruais. As alterações em mulheres não atletas também serão investigadas.

## Métodos

## Recrutamento de Participantes

Quarenta e seis mulheres saudáveis, sendo 24 atletas profissionais (idade: 20,3 ± 1,28 anos, índice de massa corporal [IMC]: 21,9 ± 2,6) com mais de 6 a 7 anos de experiência em basquete, *netball* , futsal, natação e handebol, membros da seleção nacional, e 22 não atletas (idade: 21,7 ± 2,27 anos, IMC: 22,2 ± 3,42) que não usavam contraceptivos hormonais e apresentavam ciclos regulares por 3 meses consecutivos se voluntariaram para participar deste estudo. Os critérios de inclusão foram a ausência de histórico de cirurgia no joelho ou histórico de lesão ou dor crônica nos dois membros inferiores no último ano. As participantes não tomavam nenhum medicamento específico. Mulheres amenorreicas, oligomenorreicas e polimenorreicas foram excluídas. Também excluímos as participantes submetidas a cirurgias, com traumatismo em membros inferiores e que não apresentavam ciclo menstrual regular. As participantes foram informadas detalhadamente sobre o estudo a partir da documentação fornecida e assinaram o termo de consentimento livre e esclarecido. Este estudo foi registrado (Número de Ética Médica 1010.90) e aprovado à revisão ética do *Medical Center Board* da *Malaya University* . Os métodos foram executados de acordo com as diretrizes aprovadas pelo comitê de ética institucional do *Medical Center Board* .

## Determinação da Temperatura Corporal Basal (TCB) e das Fases do Ciclo Menstrual

A temperatura corporal basal (TCB) foi usada para identificar a ovulação, caracterizada por um leve aumento da temperatura corporal central (∼0,5 ^°^ C) no meio do ciclo menstrual. ^18^ A TCB foi aferida em repouso. As fases do ciclo menstrual de cada participante foram definidas com base na TCB. A TCB correspondeu à temperatura retal (termômetro ADC, ADTemp V Fast Read Pen Type Digital, American Diagnostic, Estados Unidos). A temperatura foi medida às 7 horas da manhã durante 1 mês. Todas as participantes apresentaram ciclo ovulatório. Os resultados de TCB são mostrados na Fig. 1 .

**Fig. 1:** Gráfico de temperaturas corporais basais.

1. 36,8
2. 36,6
3. 36,4
4. 36,2
5. 35,8
6. 35,6
7. Menstruação
8. Fase folicular
9. Ovulação
10. Fase lútea
11. Gráfico de Temperatura Corporal Basal e Muco Cervical

## Medida da Composição Corporal por Análise de Impedância Bioelétrica Corporal (BIA)

A BIA é um método confiável e acessível para triagem de gordura corporal. O equipamento de BIA (BC-418, Tanita Corp., Tóquio, Japão), com placas de aço inoxidável, é usado para medida impedância de corpo inteiro e segmentar. As participantes ficaram descalças na base e seguram as alças com eletrodos embutidos; as solas de ambos os pés ficaram em contato com as placas dos eletrodos. As atletas e não atletas foram pesadas e as seguintes medidas de impedância foram obtidas: água corporal total, proteína total, mineral total, massa muscular esquelética, massa de gordura corporal, índice de massa corporal, razão cintura-quadril, pontuação de condicionamento físico (escore de condicionamento físico estimado com base na condição corporal total) e taxa metabólica basal taxa (sem clicar no equipamento, independente do grupo da participante). As medidas foram realizadas pela manhã, antes do desjejum (das 7h30 às 8h30) no mesmo dia, pelo mesmo examinador. As participantes foram instruídas a não comer ou beber antes da análise. Nas 24 horas anteriores, também não consumiram nenhum medicamento (inclusive álcool e cafeína) ou realizaram atividade física de alta intensidade que pudesse afetar os resultados.

## Coleta de Amostras de Sangue e Análises dos Níveis Séricos de Hormônios

Amostras de sangue foram coletadas três vezes em um mês: na fase folicular, na fase ovulatória e no meio da fase lútea. 19 As três fases foram determinadas por meio do gráfico de fertilidade e, nos dias indicados, as amostras de sangue foram coletadas por punção venosa. O sangue foi coletado em tubo separador (SST) e deixado para coagular em temperatura ambiente por 30 minutos. O sangue coagulado foi, então, centrifugado a 3.000 g por 15 minutos. As amostras de soro foram aliquotadas e armazenadas a -20 °C. Essas amostras foram analisadas em duplicata para a determinação da concentração de relaxina usando o *kit* de ensaio imunoenzimático (ELISA) de peptídeo específico de relaxina humana (CUSABIO, Estados Unidos, com faixa de detecção de 6,25 pg/mL a 400 pg/mL). O ELISA foi realizado de acordo com as diretrizes do fabricante. A absorbância de relaxina foi determinada com leitor de microplacas (iMark; Bio-Rad, Hercules, CA, Estados Unidos) em comprimento de onda de 450 nm. Um conjunto de diluições em série do padrão de concentrações conhecidas de relaxina foi fornecido pelo fabricante e utilizado para construção da curva-padrão para determinação dos níveis hormonais (400, 200, 100, 50, 25, 12,5, 6,25, 0 pg/mL O radioimunoensaio (RIA) foi utilizado para determinar os níveis séricos de estrógeno em pg/mL, progesterona em ng/mL e testosterona em ng/dL.

## Movimento de Rotação do Joelho

Testes de estresse em varo e valgo foram realizados no joelho para determinação da lassidão medial e lateral. As medidas foram realizadas manualmente com um goniômetro ortopédico (modelo MR0104, feito em PVC, 180°) e repetidas para confirmação em cadeira eletrônica feita sob medida, projetada como já descrito. 20 Para estabelecimento de confiabilidade, o exame foi realizado por dois avaliadores (um fisioterapeuta e um cientista do movimento humano), ambos treinados por um clínico, de forma independente. Os examinadores desconheciam a fase do ciclo menstrual das participantes. Os dois joelhos das participantes foram avaliados quanto a alterações nos ângulos de varo e valgo. O teste foi feito fixando o joelho em 0° e 20° de flexão. Lesões ligamentares não foram observadas na presença de varo/valgo ou carga axial até 20°; 21 por isso, esses ângulos foram escolhidos para este estudo. Todos os procedimentos foram idênticos aos descritos no tratado de exame clínico 22 e por van der Esch et al. 20

As medidas em varo e valgo foram realizadas manualmente com goniômetro ortopédico. O teste de varo consiste na aplicação de uma força de estresse do lado medial pela adução do tornozelo. O exame do LCL foi realizado com o joelho em 0° ou 20° de flexão e as participantes deitadas. Uma mão do examinador foi colocada sobre a linha articular lateral, enquanto a outra mão segurava a parte inferior da perna firmemente no tornozelo. O teste de valgo consiste na aplicação de uma força de estresse no lado lateral pela abdução do tornozelo. O alongamento do LCM foi realizado com o joelho em 0° ou 20° de flexão e a participante deitada. A mão do examinador foi colocada sobre a linha articular medial e a outra mão segurava firmemente a parte inferior da perna. As diferenças de ângulo foram registradas manualmente por meio de um goniômetro ortopédico.

As medidas de varo/valgo foram confirmadas em cadeira eletrônica personalizada. O intervalo de confiabilidade do teste do coeficiente de correlação intraclasse (ICC) foi realizado para detecção de diferenças mínimas nos desvios em varo e valgo ou medida de alta precisão com alta reprodutibilidade. A confiabilidade foi determinada em dez participantes, atletas e não atletas, e variou de 0,95 a 0,93 (ICC), respectivamente. As participantes sentaram-se confortavelmente na cadeira eletrônica com apoio para as costas. A articulação do joelho foi fixada em 0° ou 20° de flexão durante todo o exame. A coxa, a perna e o tornozelo foram fixados à cadeira em 0° ou 20°, impedindo os movimentos rotacionais mediais, laterais, internos e externos da perna. O pé e o membro inferior da participante foram presos com braçadeiras no tornozelo ou na parte distal da perna. A parte inferior da perna e a parte superior da coxa foram presas ao dispositivo usando uma bandagem de velcro. Um braço de movimento livre foi colocado diretamente sob a articulação tibiofemoral como eixo de rotação (meio da fossa poplítea). Para movimentação estável do joelho a 7,7 Nm, utilizou-se um peso preso por uma corda ao braço livre. A corda foi presa a 74 cm do eixo de rotação do braço. A carga foi aplicada nos lados medial e lateral da perna, resultando na medida em varo/valgo da articulação do joelho. Os avaliadores sentaram-se atrás das participantes e aplicaram a carga lentamente na parte inferior da perna com as mãos, de maneira padronizada. Um sistema de medida digital acoplado registrou o ponto final do movimento rotacional em varo/valgo durante 5 segundos. Para evitar o aumento do tônus muscular decorrente da dor durante a medida, as participantes foram instruídas a relaxar e relatar o início da dor. O movimento de lassidão do ângulo da articulação do joelho foi calculado como a soma dos desvios em varo/valgo em graus. 23 24 Os resultados dos testes manuais de lassidão do joelho foram quase iguais aos obtidos com a cadeira eletrônica.

## Análise Estatística

Todos os dados foram apresentados como média ± desvio-padrão. Os níveis hormonais e os ângulos de rotação de cada participante em diferentes fases do ciclo menstrual foram analisados por estatísticas descritivas. A igualdade de suposição de variável de Levene foi realizada e os resultados não revelaram diferenças significativas entre as observações. As médias dessas observações foram computadas e usadas para análise de dados. A análise de variância bicaudal (ANOVA) comparou os grupos de atletas e não atletas. O coeficiente de correlação de Pearson foi aplicado às variáveis com distribuição normal e o coeficiente de correlação de Spearman foi aplicado às variáveis com distribuição não normal. As análises foram feitas em SPSS 18.0 e *p* < 0,05 foi considerado estatisticamente significativo.

## Resultados

## Análises da Composição Corporal

As diferenças entre a composição corporal em atletas e não atletas em diferentes fases do ciclo menstrual são mostradas na Tabela 1 . A água corporal total média, proteína total, mineral total, massa muscular esquelética, massa de gordura corporal, índice de massa corporal, razão cintura-quadril, pontuação de condicionamento físico e taxa metabólica basal em atletas e não atletas foram apresentados. As medidas foram realizadas três vezes e os valores médios foram inseridos como valores finais. A suposição de igualdade de variável de Levene determinou que não havia diferença significativa entre essas variáveis. 25

**Tabela 1:** Características e dados demográficos das participantes em diferentes fases do ciclo menstrual

Variáveis	Atletas Média ± DP	Não atletas Média ± DP
**Idade (anos)**	20,3 ± 1,28	21,7 ± 2,27
**Altura (cm)**	163 ± 2,75	158,5 ± 5,28
**Peso (kg)**		
- **Fase folicular**	58 ± 7,93	56,5 ± 9,96
- **Fase ovulatória**	57,5 ± 7,75	55,7 ± 9,99
- **Fase lútea**	58 ± 7,93	56,1 ± 10
**Teor corporal total de água (kg)**		
- Fase folicular*	30,8 ± 2,42	27 ± 3,19
- Fase ovulatória*	30,4 ± 2,33	27 ± 3,1
- Fase lútea*	30,6 ± 2,45	27,1 ± 3,2
**Proteína total (kg)**		
- Fase folicular*	7,18 ± 0,75	8,24 ± 0,66
- Fase ovulatória*	7,21 ± 0,81	8,24 ± 0,66
- Fase lútea*	7,21 ± 0,85	8,22 ± 0,65
**Mineral total (kg)**		
- Fase folicular*	3,08 ± 0,25	2,73 ± 0,32
- Fase ovulatória*	3,03 ± 0,26	2,69 ± 0,3
- Fase lútea*	3,05 ± 0,27	2,71 ± 0,33
**Massa muscular esquelética (kg)**		
- Fase folicular*	23 ± 2,05	19,7 ± 2,55
- Fase ovulatória*	22,7 ± 2,02	19,7 ± 2,47
- Fase lútea*	22,8 ± 1,91	19,8 ± 2,58
**Massa corporal de gordura (kg)**		
- Fase folicular*	15,7 ± 5,34 (26,7%)	19,1 ± 7,02(33,4%)
- Fase ovulatória*	15,9 ± 5,44 (26,7%)	19 ± 7,34 (33%)
- Fase lútea*	16,1 ± 5,5 (27,3%)	19 ± 7,06 (33%)
** Índice de massa corporal (kg/m ^2^ ) **		
- **Fase folicular**	21,9 ± 2,55	22,2 ± 3,37
- **Fase ovulatória**	21,8 ± 2,59	22,2 ± 3,51
- **Fase lútea**	22 ± 2,67	22,2 ± 3,39
Razão quadril-cintura*	0,82 ± 0,04	0,84 ± 0,06
Pontuação de condicionamento físico (pontos)*	75,1 ± 2,93	69,3 ± 5,51
**Taxa metabólica basal (kcal)**		
- Fase folicular*	1284 ± 75,8	1160 ± 96,6
- Fase ovulatória*	1272 ± 75	1167 ± 90,9
- Fase lútea*	1275 ± 77,2	1170 ± 94,7

Os dados correspondem à média ± desvio-padrão (DP) das medidas de impedância. Existem diferenças estatisticamente significativas na maioria das variáveis entre o grupo de atletas e não atletas (*p< 0,05).

* *p* < 0,05 à comparação entre os grupos de atletas e não atletas.

Nossos achados indicaram que o peso médio das atletas foi maior do que o das não atletas, mas sem diferenças estatisticamente significativas. O peso foi maior na fase lútea e durante a menstruação em comparação à fase folicular. De modo geral, as atletas tinham maior teor de água corporal total em comparação a não atletas. No entanto, não houve diferenças significativas na água corporal total entre as fases do ciclo menstrual. Os valores de proteína total foram significativamente maiores em não atletas em comparação a atletas ( *p* < 0,05) e nenhuma diferença significativa foi observada entre as fases do ciclo menstrual. O teor mineral total em atletas foi significativamente maior do que em não atletas e, em ambos os grupos, foi maior durante a menstruação.

A massa muscular esquelética foi maior em atletas em comparação a não atletas ( *p* < 0,05) e não houve nenhuma diferença significativa entre as fases do ciclo menstrual. A massa de gordura corporal foi maior em não atletas do que em atletas ( *p* < 0,05) e nenhuma diferença significativa foi observada entre as fases do ciclo menstrual. O índice de massa corporal foi ligeiramente menor em atletas do que em não atletas, sem diferença significativa entre os dois grupos. A razão cintura-quadril foi significativamente menor em atletas do que em não atletas, enquanto a pontuação de condicionamento físico foi significativamente maior em atletas do que em não atletas (p < 0,05). Por fim, as atletas apresentaram maior TMB do que as não atletas, mas sem nenhuma diferença significativa entre as fases do ciclo menstrual.

## Ângulos da Articulação do Joelho nas Diferentes Fases do Ciclo Menstrual

As Tabelas 2 e 3 mostram os ângulos do joelho em 0° e 20° de flexão em testes de estresse em varo e valgo em atletas e não atletas. Nos testes de flexão de 0° e 20° em varo, as não atletas apresentam maior ângulo do joelho em relação às atletas em todas as fases do ciclo. Em ambos os grupos, o ângulo do joelho foi maior na fase lútea, seguido da fase folicular e, por fim, da fase ovulatória. Nos testes de flexão de 0° e 20° em valgo, as não atletas parecem ter maior ângulo do joelho em todas as fases do ciclo em comparação às atletas ( *p* < 0,05). O maior ângulo foi observado na fase lútea, seguida da fase folicular e da fase ovulatória.

**Tabela 2:** Ângulos da articulação do joelho de atletas e não atletas (obtidos manualmente com goniômetro ortopédico)

	Atletas Média (DP)	Não atletas Média (DP)	valor de *t*	Valor de *p*	Tamanho do efeito
**Varo 0°**					
- **Fase folicular**	3,75 (1,13)	5,33(1,02)	4,63	<0,05	1,47
- **Fase ovulatória**	1,80 (0,43)	3,44(0,95)	7,00	<0,05	2,38
- **Fase lútea**	5,45 (1,01)	7,19(1,01)	5,46	<0,05	1,72
**Valgo 0°**					
- **Fase folicular**	3,09 (1,21)	4,76(0,89)	4,99	<0,05	1,60
- **Fase ovulatória**	1,39 (0,51)	3,44(0,56)	12,09	<0,05	3,83
- **Fase lútea**	4,76 (1,32)	5,56(0,72)	2,37	<0,05	0,78
**Varo 20°**					
- **Fase folicular**	11,48(1,21)	12,80(1,03)	3,74	<0,05	1,18
- **Fase ovulatória**	9,35(0,75)	10,95(0,72)	6,89	<0,05	2,18
- **Fase lútea**	14,25(1,18)	15,11(1,05)	2,44	<0,05	0,77
**Valgo 20°**					
- **Fase folicular**	9,58(1,56)	11,71(1,51)	4,40	<0,05	1,39
- **Fase ovulatória**	7,63(1,00)	10,11(0,88)	8,36	<0,05	2,64
- **Fase lútea**	11,64(1,29)	12,98(1,79)	2,73	<0,05	0,87

Os ângulos de varo/valgo da articulação do joelho em 0 e 20° de atletas e não atletas são apresentados como média ± desvio-padrão (DP). Há diferenças estatisticamente significativas entre atletas e não atletas (p< 0,05). Os resultados indicam que não atletas apresentavam maior ângulo do joelho em comparação a atletas em três fases diferentes do ciclo menstrual (menstruação, fases folicular e lútea). O tamanho do efeito é > 0,8.

**Tabela 3:** Ângulos da articulação do joelho de atletas e não atletas (confirmados com o uso de cadeira eletrônica personalizada)

	Atletas Média (DP)	Não atletas Média (DP)
**Varo 0°**		
- **Fase folicular**	3,88 (1,57)	5,52(1,77)
- **Fase ovulatória**	1,49 (0,71)	4,01(1,53)
- **Fase lútea**	4,95 (1,32)	6,97(1,68)
**Valgo 0°**		
- **Fase folicular**	3,22 (1,18)	4,64(0,52)
- **Fase ovulatória**	1,49 (1,32)	3,81(1,62)
- **Fase lútea**	4,69 (1,76)	6,03(1,25)
**Varo 20°**		
- **Fase folicular**	10,81(3,02)	11,98(2,11)
- **Fase ovulatória**	9,73(2,41)	10,83(1,56)
- **Fase lútea**	13,55(3,12)	14,87(1,55)
**Valgo 20°**		
- **Fase folicular**	9,85(1,56)	11,59(2,31)
- **Fase ovulatória**	7,71(2,07)	10,67(1,05)
- **Fase lútea**	12,02(2,14)	12,69(2,13)

## Alterações nos Níveis Séricos de Esteroides Sexuais e Relaxina nas Diferentes Fases do Ciclo Menstrual

A Tabela 4 mostra os níveis séricos de esteroides sexuais e relaxina em atletas e não atletas. Nossos achados indicam que o maior nível de estrógeno foi observado na fase ovulatória em ambos os grupos, sem diferenças significativas entre atletas e não atletas. Os níveis de progesterona foram mais altos na fase lútea e as atletas apresentaram valores significativamente menores do que as não atletas ( *p* < 0,05). Da mesma forma, o nível de progesterona também foi maior em não atletas em comparação às atletas na fase folicular, embora esse nível tenha sido cerca de 12 vezes menor do que na fase lútea do ciclo. Nenhuma diferença significativa nos níveis de progesterona foi observada na fase ovulatória entre os dois grupos.

**Tabela 4:** Níveis de esteroides sexuais e relaxina em diferentes fases do ciclo menstrual

	Atletas Média (DP)	Não atletas Média (DP)	valor de *t*	Valor de *p*	Tamanho do efeito
**Estrógeno (pg/mL)**					
- **Fase folicular**	170,20 (39,86)	197,65 (71,97)	1,49	>0,05	0,50
- **Fase ovulatória**	507,35 (161,52)	492,30 (276,96)	0,21	>0,05	0,07
- **Fase lútea**	439,50 (150,54)	409,45 (163,89)	0,60	>0,05	0,19
**Progesterona (ng/mL)**					
- **Fase folicular**	2,28 (1,00)	4,19 (2,53)	3,14	<0,05 **	1,08
- **Fase ovulatória**	1,80 (1,24)	1,74 (0,60)	0,20	>0,05	0,07
- **Fase lútea**	24,74 (11,34)	31,33 (8,09)	2,12	<0,05 **	0,68
**Testosterona (ng/mL)**					
- **Fase folicular**	0,82 (0,38)	1,24 (0,47)	3,08	<0,05 **	0,99
- **Fase ovulatória**	1,21 (0,54)	1,39 (0,48)	1,12	>0,05	0,36
- **Fase lútea**	1,22 (0,61)	1,37 (0,63)	0,76	>0,05	0,24
**Relaxina (pg/mL)**					
- **Fase folicular**	2,10 (0,56)	1,69 (1,27)	1,33	>0,05	0,45
- **Fase ovulatória**	1,38 (0,87)	0,34 (0,45)	4,73	<0,05 **	1,58
- **Fase lútea**	15,58 (5,36)	10,35 (2,96)	3,82	<0,05 **	1,26

Os níveis de esteroides sexuais e relaxina são apresentados em diferentes fases do ciclo menstrual como média ± desvio-padrão (DP).** *p* < 0,05 (comparação entre atletas e não atletas). O tamanho do efeito é > 0,8.

Os níveis de testosterona, que eram mais baixos do que os de estrógeno e a progesterona, foram maiores em não atletas do que em atletas. Nas participantes não atletas, os níveis de testosterona foram maiores na fase ovulatória, seguida pela fase lútea. Os menores níveis de testosterona foram observados em atletas na fase folicular. Os níveis séricos de relaxina foram mais elevados em atletas em comparação a não atletas, em particular durante as fases ovulatória e lútea do ciclo. Não foram observadas diferenças significativas nos níveis de relaxina durante a fase folicular entre os dois grupos.

## Correlações entre os Níveis de Esteroides Sexuais e Relaxina nos Ângulos da Articulação do Joelho

As Tabelas 5 , 6 e 7 mostram a correlação entre os níveis de esteroides sexuais e os ângulos da articulação do joelho em mulheres atletas e não atletas. De modo geral, foram observadas fortes correlações entre os níveis de progesterona e relaxina e os ângulos articulares do joelho nos testes de varo e valgo (0° e 20° de flexão), tanto em atletas quanto em não atletas. As maiores correlações entre os níveis séricos de progesterona e relaxina e os ângulos do joelho foram detectadas no teste de varo em 20° de flexão em atletas e não atletas.

**Tabela 5:** Coeficiente de correlação entre esteroides sexuais e ângulos do joelho

	0 ^o^		20 ^o^	
	varo	valgo	varo	valgo
**Estrógeno** - **Pearson**	-0,15	-0,17	-0,09	-0,14
**Progesterona** - **Spearman**	0,70**	0,62**	0,76**	0,62**
**Testosterona** - **Pearson**	0,07	0,12	0,12	0,07
**Relaxina** - **Spearman**	0,58**	0,50**	0,65**	0,47**

**Tabela 6:** Coeficiente de correlação entre esteroides sexuais e ângulos do joelho em atletas

	0 ^o^		20 ^o^	
	varo	valgo	varo	valgo
**Estrógeno** - **Pearson**	-0,13	-0,15	-0,04	-0,14
**Progesterona** - **Spearman**	0,66**	0,58**	0,70**	0,59**
**Testosterona** - **Pearson**	0,05	0,03	0,06	-0,06
**Relaxina** - **Spearman**	0,69**	0,65**	0,74**	0,64**

**Tabela 7:** Coeficiente de correlação entre esteroides sexuais e ângulos do joelho em não atletas

	0 ^o^		20 ^o^	
	varo	valgo	varo	valgo
**Estrógeno** - **Pearson**	-0,17	-0,26*	-0,13	-0,17
**Progesterona** - **Spearman**	0,72**	0,65**	0,80**	0,60**
**Testosterona** - **Pearson**	-0,10	0,01	0,01	0,00
**Relaxina** - **Spearman**	0,81**	0,73**	0,80**	0,62**

*A correlação é significativa em *p* < 0,05.

**A correlação é significativa em *p* <0,01.

## Discussão

Os principais achados deste estudo são (i) atletas têm menor grau de lassidão medial e lateral do joelho em comparação a não atletas; (ii) atletas e não atletas apresentam maior lassidão medial e lateral do joelho na fase lútea do ciclo; (iii) os níveis de progesterona e relaxina em atletas e não atletas foram mais altos na fase lútea, enquanto os níveis de estrógeno foram mais altos na fase ovulatória; (iv) os níveis de progesterona nas fases lútea e folicular foram menores em atletas em comparação a não atletas; e (v) houve forte correlação entre os níveis séricos de progesterona e relaxina e lassidão medial e lateral do joelho (em varo e valgo a 0 e 20°) em atletas e não atletas. Outros achados são que as atletas têm maior teor de água corporal total e mineral total, massa muscular esquelética, pontuação de condicionamento físico e TMB em comparação a não atletas. O teor total de proteína, a massa de gordura corporal e a razão cintura-quadril em atletas, porém, foram menores do que em não atletas.

A lassidão medial e lateral do joelho foi maior na fase lútea, o que é correlacionado aos maiores níveis séricos de progesterona e relaxina. O aumento da lassidão medial e lateral pode predispor a instabilidades do joelho, elevando, portanto, o risco de luxação do joelho e de lesões sem contato nessa articulação, como a ruptura de ligamento e menisco. Relatos anteriores revelaram que a lassidão anterior do joelho aumenta conforme os níveis de progesterona. 26 Incidências maiores de ruptura do LCA e lesão sem contato no joelho foram relatadas nas fases pós-ovulatória ou lútea, onde os níveis de progesterona eram altos. 8

Recentemente, foi relatado que a lassidão anterior e o movimento em valgo do joelho são correlacionados aos níveis de progesterona no sangue. 27 Em animais, como ratos, a lassidão do LCL foi maior nas fases de diestro e proestro, sendo correlacionada aos altos níveis séricos de progesterona. 28 As evidências sugerem que a influência da progesterona na lassidão do joelho pode ser decorrente do aumento da degradação do colágeno, pois os níveis séricos de biomarcadores de degradação do colágeno eram altos no início da fase lútea do ciclo menstrual, como relatado em mulheres eumenorreicas. 29 Nossos achados em atletas indicando aumento da lassidão do joelho na fase lútea e correlacionado aos níveis séricos elevados de progesterona estão de acordo com o relato de Heitz et al., 30 que descreveram um aumento significativo na lassidão do LCA no pico dos níveis de progesterona em mulheres fisicamente ativas.

Neste estudo, os níveis séricos de relaxina foram correlacionados à lassidão do joelho. Os níveis séricos de relaxina foram mais elevados na fase lútea e paralelos ao aumento dos níveis séricos de progesterona. Isso condiz com um relato em atletas do sexo feminino em que o nível de relaxina sérica é positivamente correlacionado ao nível sérico de progesterona. 31 Em atletas, o nível sérico de relaxina durante a fase ovulatória e lútea foi cerca de 4,0 e 1,5 vezes maior, respectivamente, em comparação a não atletas. Ainda em atletas, os níveis séricos de relaxina foram mais altos na fase lútea (15,58 ± 5,36 pg/mL), sugerindo que essa molécula pode influenciar a lassidão medial e lateral do joelho. Portanto, o aumento do nível sérico de relaxina pode contribuir para o aumento da lassidão medial e lateral do joelho, como observado em atletas e não atletas durante a fase lútea do ciclo. Nossos achados foram apoiados por um estudo de Dragoo et al. 32 (2011) relatando que os níveis séricos de relaxina são fortemente correlacionados à incidência de ruptura do LCA em atletas universitárias de elite. Em estudos com animais, a administração de relaxina a cobaias aumentou a lassidão do LCA. O aumento da lassidão pode ser devido a vários fatores, inclusive a regulação positiva de metaloproteases da matriz (MMPs) 33 que degradam o colágeno. 34 Além disso, a progesterona também pode aumentar a ação da relaxina por meio da regulação positiva da expressão de isoformas dos receptores de relaxina RXFP1 e RXFP2 nos ligamentos colaterais do joelho e no tendão patelar de ratos. 35 O receptor de relaxina também foi expresso no LCA de roedores 36 e nas articulações carpometacárpicas de humanos. 37

Este estudo não detectou diferenças significativas nos níveis de estrógeno e testosterona entre atletas e não atletas nas fases lútea e ovulatória do ciclo menstrual. Em atletas, o nível sérico de testosterona foi ligeiramente menor na fase folicular. Embora os níveis séricos de estrógeno fossem maiores na fase ovulatória, a lassidão parecia ser menor. Portanto, concluímos que o alto nível de estrógeno pode não ser responsável pelo aumento da lassidão medial e lateral do joelho. Nossos achados contrastam com vários outros relatos que indicam que altos níveis séricos de estrógeno foram responsáveis pelo aumento da incidência de lesão sem contato no joelho durante a fase ovulatória. 12 38 39 As diferenças entre nossos achados e outros não eram claras. A menor lassidão do joelho na fase ovulatória sugere que altos níveis de estrógeno podem diminuir o risco de lesão sem contato no joelho, diferentemente dos altos níveis séricos de progesterona, que têm o efeito oposto. Nossos achados apoiaram os de Wojtys et al, 8 que observaram um número significativamente menor de lesões não traumáticas do joelho na fase folicular em comparação à fase pós-ovulatória. Alternativamente, o menor risco de lesão não traumática do joelho na fase ovulatória pode ser decorrente dos menores níveis séricos progesterona e relaxina.

Neste estudo, a testosterona pode não ter influenciado a lassidão medial e lateral do joelho, pois não houve correlação positiva entre o nível sérico dessa molécula e os parâmetros do joelho. Nenhuma diferença significativa nos níveis séricos de testosterona foi observada entre atletas e não atletas nas fases ovulatória e lútea do ciclo menstrual. Um estudo recente de O'Leary et al. 40 relatou que o exercício aeróbico prolongado em mulheres com ciclo menstrual normal pode induzir uma elevação de curto prazo no nível plasmático de testosterona. Este efeito, no entanto, não foi observado em nosso estudo; entretanto, relatamos que o efeito da testosterona na lassidão do joelho pode ser mascarado pelo efeito do estrógeno e da progesterona.

Em conclusão, demonstramos que as alterações na lassidão medial e lateral do joelho em atletas e não atletas diferem conforme as fases do ciclo menstrual. Mostramos que os hormônios sexuais séricos, em particular a progesterona, têm forte correlação positiva com a lassidão medial e lateral do joelho. Wiertsema et al. 41 relataram que exames clínicos, como o teste de Lachman, são confiáveis para a determinação da lassidão anteroposterior do LCA. Embora o artrômetro possa ser usado para análise do movimento tibiofemoral anterior, tem aplicação limitada ao medir a lassidão medial e lateral do joelho. A diminuição observada na lassidão do joelho em atletas pode ser devido a um maior controle muscular, pois essas participantes têm maior massa muscular, nível de condicionamento físico e diferenças nas propriedades biomecânicas do ligamento devido à diferença de carga. 42 A maior massa muscular contribuiria para um maior tônus que resiste ao movimento da articulação do joelho. A maior lassidão medial e lateral do joelho em não atletas, especialmente na fase lútea, sugere que esse grupo é mais suscetível a lesões não traumáticas nesta articulação.

## Conclusão

Os achados deste estudo são importantes para o campo da fisiologia e medicina esportiva, pois podem indicar o mecanismo subjacente ao aumento da incidência de lesões não traumáticas no joelho observadas em mulheres durante a fase lútea do ciclo menstrual. Diante disso, cuidados são necessários para reduzir o risco de lesão não traumática do joelho em atletas do sexo feminino durante esta fase do ciclo.
